# Predictive value of postoperative day 1 parathyroid hormone levels for early and late hypocalcaemia after thyroidectomy

**DOI:** 10.1007/s00423-022-02480-1

**Published:** 2022-03-05

**Authors:** Fiona Riordan, Catherine Brophy, Matthew S. Murphy, Patrick Sheahan

**Affiliations:** 1 Dept Otolaryngology, Head and Neck Surgery, South Infirmary Victoria University Hospital, Cork, Ireland; 2 Dept Endocrinology, South Infirmary Victoria University Hospital, Cork, Ireland; 3 Dept Surgery, University College Cork, Cork, Ireland; 4 ENTO Research Unit, University College Cork, Cork, Ireland

**Keywords:** Parathyroid hormone, Thyroidectomy, Hypoparathyroidism, Hypocalcemia

## Abstract

**Purpose:**

Early parathyroid hormone (PTH) levels after total thyroidectomy can predict patients at low risk of hypocalcaemia who can be discharged early without calcium supplementation. For centres without facility to perform early PTH levels, PTH levels sent on the first postoperative day (POD1) may be an alternative. However, there is less data regarding optimal cut-off PTH levels for POD1 discharge.

**Methods:**

Retrospective review of prospective database of thyroid operations between September 2009 and February 2020 at tertiary referral centre. Main outcome measure was symptomatic hypocalcaemia.

**Results:**

Five hundred seventy patients undergoing total (521) or completion thyroidectomy with POD1 PTH levels available were included. Among patients with POD1 PTH levels ≥ 20 pg/ml and POD1 calcium ≥ 2.0 mmol/l, the incidence of symptomatic hypocalcaemia was 1% (3/300), and need for intravenous calcium 0.3% (1/300). For POD1 PTH levels 15–19 pg/ml and POD1 calcium ≥ 2.0 mmol/l, the incidence of symptomatic hypocalcaemia and need for intravenous calcium was 5.4% (3/55). For PTH levels 10–14 pg/ml and calcium ≥ 2.0 mmol/l, the incidence of symptomatic hypocalcaemia and need for intravenous calcium was 11.7% (7/60). The risk of permanent hypoparathyroidism was < 1% for POD1 PTH levels ≥ 15 pg/ml; 5.4% for levels 10–14 pg/ml; and 19.8% for levels < 10 pg/ml.

**Conclusions:**

POD1 PTH levels ≥ 15 pg/ml along with calcium ≥ 2.0 mmol/l are associated with low risk of symptomatic hypocalcaemia, and represent a safe criterion for discharge of most patients without calcium supplementation. For certain patient groups, a higher threshold of 20 pg/ml could be considered.

## Introduction

Hypocalcaemia after thyroidectomy is believed to arise from functional disruption of parathyroid glands during surgery. Symptomatic hypocalcaemia, which may require treatment with intravenous calcium, is reported after 10–36% of total thyroidectomies[1–6]. Because onset of hypocalcaemia can be delayed for 1–2 days postoperatively, traditional management has been to keep patients in hospital for two or more postoperative nights for monitoring of calcium levels, or alternatively to discharge with prophylactic calcium supplementation[1, 2].

Because of the very short half-life of parathyroid hormone (PTH), PTH levels performed at earlier time points after surgery can be used as an early predictor of post-thyroidectomy hypocalcaemia, and thus to select patients who can be safely discharged from hospital earlier without calcium supplementation[4, 5, 7–12]. Most of the data supporting early PTH levels comes from studies based on PTH measurements taken within 4–6 h of surgery[4, 5, 7–10, 12]. In centres without facility to perform early PTH levels, PTH levels sent on the morning of the first postoperative day (POD1) may be a good alternative[13–17]. However, not all authors are in agreement that POD1 PTH levels reliably predict hypocalcaemia[2], while cut-off PTH levels on POD1 for safe patient discharge are not clearly defined[18].

The purpose of the present study was to examine utility of POD1 PTH levels as predictor of post-thyroidectomy symptomatic hypocalcaemia at our institution, and to determine cut-off levels for selection of patients for early discharge.

## Methods

Ethical approval was obtained from the University Clinical Research Ethics Committee. The study comprised a retrospective review of a prospectively maintained thyroid surgery database, review of electronic laboratory information system, and retrospective chart review. Inclusion criteria were consecutive patients undergoing total or completion total thyroidectomy between September 2009 and February 2020 at our unit, a teaching hospital, and tertiary referral centre for thyroid surgery. The thyroid database contained patient and surgical details, as well as occurrence of symptoms of hypocalcaemia during the hospital stay. Calcium and PTH levels were obtained from the electronic laboratory information system. In addition, retrospective review of charts of all patients developing hypocalcaemia was performed to check for documentation of symptoms of hypocalcaemia not captured by the database, administration of intravenous calcium, prescription of oral calcium, and, in case of patients discharged on oral calcium, verification if calcium had been stopped with return to normocalcaemia.

### Perioperative management

During the study period, our policy was to admit patients for a minimum two-night stay after total thyroidectomy. Preoperative bloods included thyroid function tests, calcium and PTH levels. Patients were not routinely prescribed calcium or vitamin D perioperatively. Postoperatively, PTH levels were sent at 6 a.m. on POD1. Calcium levels were sent at 6 a.m. and 3 p.m. on POD1, and at 6 a.m. on postoperative day 2 (POD2). PTH levels were measured using the Roche Elecsys PTH assay (Roche Diagnostics GmbH, Switzerland) between 2009 and 2017 (normal range 15–70 pg/ml), and the Abbott ARCHITECT i4000SR assay (Abbott laboratories, Ireland) between 2017 and 2020 (normal range 15–68 pg/ml). Asymptomatic patients with calcium levels remaining above 2.0 mmol/l on POD2 were discharged. The decision regarding discharge was based on calcium levels and symptoms, and not PTH levels. Patients developing symptoms of hypocalcaemia were kept in hospital and treated with intravenous calcium as required, and commenced on oral calcium. Asymptomatic patients with below normal calcium levels on POD2 were commenced on oral calcium, and/or kept in hospital for additional day(s) until the trajectory in calcium levels was upwards.

### Definitions

Biochemical hypocalcaemia was defined as any postoperative laboratory calcium measurement < 2.0 mmol/l. The normal calcium range at our institution is 2.10–2.65 mmol/l; however, we have used the 2.0 mmol/l cut-off as symptoms of hypocalcaemia are rare above this threshold. Symptomatic hypocalcaemia was defined as any symptoms of paraesthesia, carpopedal spasm, or positive Chvostek’s or Trousseau’s sign, at any point during the hospital stay. Permanent hypoparathyroidism was defined as any calcium level < 2.10 mmol/l > 6 months postoperatively, or any need for calcium and/or vitamin D > 6 months postoperatively, irrespective of PTH or calcium levels.

### Statistical analysis

The incidence of development of biochemical or symptomatic hypocalcaemia at any point in the postoperative period was described according to POD1 PTH and calcium measurements. Differences between patient groups according to POD1 PTH levels were analyzed using Fisher’s exact test.

## Results

During the study period, 632 patients underwent total or completion total thyroidectomy. Five hundred seventy had POD1 PTH levels available and comprised the final study population. Table 1 shows demographic and clinical characteristics of the entire study group, as well as subgroups defined by POD1 PTH levels below or above the lower institutional limit (15 pg/ml). There was a higher incidence of low POD1 PTH levels among patients undergoing total (versus completion) thyroidectomy, central neck dissection, having cancer diagnosis, and with parathyroid tissue identified on surgical pathology.

**Table 1:** Clinical and demographic features of the study group

	Entire cohort (*n* = 570)	POD1 PTH < 15 pg/ml (*n* = 197)	POD1 PTH ≥ 15 pg/ml (*n* = 373)	*p*-value
Male (versus female)	83 (14.6%)	23 (11.7%)	60 (16.1%)	0.17
Age (mean, years)	50.4	49.5	50.9	0.3
Total thyroidectomy (versus completion)	521 (91.4%)	187 (94.9%)	334 (89.5%)	0.03
Graves disease	125 (21.9%)	36 (18.3%)	89 (23.4%)	0.14
Central neck dissection	71 (12.5%)	50 (25.4%)	21 (5.6%)	< 0.0001
Retrosternal	101 (17.7%)	35 (17.8%)	66 (17.7%)	> 0.99
Revision surgery	12 (2.1%)	4 (2.0%)	8 (2.1%)	> 0.99
Cancer diagnosis	153 (26.8%)	72 (36.5%)	81 (21.7%)	0.0002
Parathyroid in specimen	101 (17.8%)	58 (29.4%)	43 (11.5%)	< 0.0001

One hundred eighty-five patients (32.5%) developed biochemical hypocalcaemia. Seventy-six (13.3%) had calcium levels < 2.0 mmo/l at the first (6 a.m. POD1) calcium draw, the remainder developing hypocalcaemia at later time points. Ninety-three patients (16.3%) developed symptomatic hypocalcaemia, and 79 (13.9%) required intravenous calcium replacement. Of note, 2 patients with symptoms of hypocalcaemia (one of whom received intravenous calcium) had lowest measured calcium levels > 2.0 mmol/l (2.03 mmol/l and 2.05 mmol/l respectively). The incidence of biochemical and symptomatic hypocalcaemia, and need for calcium supplementation, according to POD1 PTH levels, is shown in Table 2.

**Table 2:** Incidence of hypocalcaemia, treatment with intravenous and oral calcium, and permanent hypoparathyroidism, according to POD1 PTH levels

PTH level (pg/ml)	*n*	Symptomatic hypocalcaemia	Biochemical hypocalcaemia	Intravenous calcium	Oral calcium	Permanent hypoparathyroidism
< 10	123	70 (56.9%)	105 (85.3%)	58 (47.1%)	85 (69.1%)	24 (19.5%)
10–14	74	14 (18.9%)	32 (43.2%)	14 (18.9%)	18 (24.3%)	4 (5.4%)
15–19	61	6 (9.8%)	20 (32.8%)	6 (9.8%)	8 (13.1%)	1 (1.6%)
≥ 20	312	3 (1.0%)	28 (9.0%)	1 (0.3%)	4 (1.3%)	1 (0.3%)

Table 3 shows incidence of biochemical and symptomatic hypocalcaemia and need for intravenous calcium according to PTH levels in patients who also had 6 a.m. POD1 calcium levels ≥ 2.0 mmol/l. Among patients with both POD1 PTH levels ≥ 20 pg/ml and 6 a.m. POD1 calcium levels ≥ 2.0 mmol/l, 3 (1.0%) went on to develop symptomatic hypocalcaemia, of whom one (0.3%) was treated with intravenous calcium. Among patients with POD1 PTH levels of 15–19 pg/ml, together with 6 a.m. POD1 calcium levels ≥ 2.0 mmol/l, the risk of symptomatic hypocalcaemia and need for intravenous calcium was 5.4% (3/55). Among patients with POD1 PTH levels 10–14 pg/ml and 6 a.m. POD1 calcium levels ≥ 2.0 mmol/l, the risk of symptomatic hypocalcaemia and need for intravenous calcium was 11.7% (7/60). Finally, among patients with PTH levels < 10 pg/ml and 6 a.m. POD1 calcium levels ≥ 2.0 mmol/l, the risk of symptomatic hypocalcaemia was 46.8%, and need for intravenous calcium 38%.

**Table 3:** Incidence of biochemical and symptomatic hypocalcaemia, and treatment with intravenous and oral calcium, according to POD1 PTH levels, among patients with POD1 calcium levels ≥ 2 mmol/l

Group	*n*	Symptomatic hypocalcaemia	Biochemical hypocalcaemia	Intravenous calcium
PTH level < 10 pg/ml and Ca ≥ 2.0 mmol/l	79	37 (46.8%)	61 (77.2%)	30 (38.0%)
PTH level 10–14 pg/ml and Ca ≥ 2.0 mmol/l	60	7 (11.7%)	18 (30%)	7 (11.7%)
PTH level 15–19 pg/ml and Ca ≥ 2.0 mmol/l	55	3 (5.5%)	14 (25.5%)	3 (5.5%)
PTH level ≥ 20 pg/ml and Ca ≥ 2.0 mmol/l	300	3 (1%)	16 (5.3%)	1 (0.3%)

Regarding the 6 patients with POD1 PTH ≥ 15 pg/ml and calcium ≥ 2.0 mmol/l who went on to develop symptomatic hypocalcaemia, 3 were undergoing surgery for Graves disease, one had a history of Graves disease, and one was undergoing surgery for non-Graves hyperthyroidism. However, there was no significant difference in incidence of symptomatic hypocalcaemia among patients with POD1 PTH ≥ 15 pg/ml between patients undergoing surgery for Graves disease versus other indications (*p* = 0.15).

Follow-up bloods were available for 171/185 patients with biochemical hypocalcaemia. Thirty (5.3%) patients were defined as having permanent hypoparathyroidism, based on most recent calcium levels < 2.10 mmol/l (6), or ongoing need for calcium and/or vitamin D supplementation in patients with normal calcium levels (24). Most (24/30) patients with permanent hypoparathyroidism had POD1 PTH levels < 10 pg/ml. Among patients with POD1 PTH levels 10–14 pg/ml, the risk of permanent hypoparathyroidism was 5.4%, while among patients with POD1 PTH levels 15–19 pg/ml and ≥ 20 pg/ml, the risk was 1.6% and 0.3%, respectively (Table 2).

## Discussion

Postoperative PTH levels have been reported by numerous studies to be reliable early predictors of patients at low risk of hypocalcaemia, who are hence suitable for early discharge[11]. Among published studies, the timing of PTH measurement after surgery varies, ranging from intraoperative[4, 8], early (1–4 h)[5, 10, 12, 19, 20], 6 h[7, 9], or next day[13–17]. However, not all authors are in agreement that a single postoperative PTH level is sufficient to exclude significant hypocalcaemia. In one of the largest published series (523), Lombardi reported a threshold 4-h PTH level of 10 pg/ml to have too high a high false negative to allow safe early discharge[3], while Sahli reported 1-h PTH levels to not accurately predict patients at risk of hypocalcaemia[21]. Simultaneous assessment of both early calcium and PTH levels has been suggested as being more accurate[7, 22], while Pisanu reported that combined measurement of 6-h PTH levels and POD1 calcium levels gave the best predictive outcome[9, 23].

The downside of early PTH and calcium assessment is that not all centres have access to PTH analysers and staff to perform the measurements at the specified time points, which may be outside normal working hours. Furthermore, calcium draws performed less than 6 h postoperatively may be too early to show any significant calcium drop. Among institutions without the facility to perform early postoperative PTH, measurements taken the morning after surgery may serve as an alternative to select patients for discharge on POD1[5, 13–18]. In addition, for units with a policy of keeping all thyroidectomies overnight due to concerns about the risk of haematoma, early PTH levels offer little advantage over POD1 PTH levels with respect to facilitating early discharge. However, there is little agreement among published studies regarding safe cut-off POD1 PTH levels, which may differ from early PTH cut-offs[18]. Furthermore, the authors of the single largest published study reported the predictive value of POD1 PTH levels to be poor[2].

The findings of the present study would suggest that a cut-off POD1 PTH level of 15 pg/ml, along with 6 a.m. POD1 calcium ≥ 2.0 mmol/l, is likely to be a safe criterion for discharge of most patients without need for oral calcium supplementation. This criterion would have facilitated POD1 discharge of 62% of patients in the current series. However, for those patients with PTH levels between 15 and 19 pg/ml, there was a small but not negligible risk of developing symptomatic hypocalcaemia (5.4%). Therefore, a higher PTH threshold of 20 pg/ml could be considered for certain patient groups, such as patients with difficulty understanding postoperative instructions, and/or who would have difficult returning to the hospital if they developed symptoms. It was an interesting observation that many patients with POD1 PTH ≥ 15 pg/ml who developed symptoms had a history of Graves disease or hyperthyroidism; however, in this series, we were not able to conclusively demonstrate an association between Graves disease and development of symptomatic hypocalcaemia in patients with POD1 PTH ≥ 15 pg/ml. On the other hand, among patients with POD1 PTH levels of 10–14 pg/ml and calcium levels ≥ 2.0 mmol/l, 12% developed symptoms requiring intravenous calcium. Thus, this group may benefit from supplemental calcium, and/or an extra night in hospital with further monitoring of calcium levels.

The POD1 PTH cut-offs which we report are somewhat higher than those reported in some other studies. It is possible that much of this discrepancy is related to differences in study methodology, and sample size. For example, Cayo reported POD1 PTH levels ≥ 10 pg/ml to be a safe threshold for patient discharge without calcium supplementation, as only 10% (11/112) of patients with PTH levels above this cut-off developed symptoms, all of which were managed at home without hospital readmission. However, the number of patients with PTH levels 10–14 pg/ml and incidence of symptoms in this group is not given[13]. Landry reported that calcium supplementation could be limited to patients with POD1 PTH < 6 pg/ml or calcium < 8 mg/ml. However, in this series, all patients were discharged with instructions to take calcium if they developed symptoms, and documentation of usage of oral calcium and development of symptoms were reliant on a nurse telephone follow-up[15]. Selberherr reported no cases of symptomatic hypocalcaemia with POD1 PTH levels ≥ 15 pg/ml. Taking into consideration differences in cohort size, these findings would appear to be not significantly different from the findings of the present study [16]. In a study of 101 patients, Filho reported an optimal POD1 PTH cut-off of 14.3 pg/ml, which is similar to what we found. Interestingly, the optimal PTH cut-off level on POD1 was different to the optimal cut-off 4-h PTH level (19.6 pg/ml)[18]. Finally, Kosec reported a POD1 PTH level < 2.9 pmol/l (equivalent to 27 pg/ml) to predict hypocalcaemia on the fifth postoperative day, which was similar to the optimal 1-h postoperative cut-off (< 2.99 pmol/l)[20]. Other authors reported on percentage decrease in PTH values rather than absolute levels[14], and/or used biochemical rather than symptomatic hypocalcaemia as the main outcome measure[14, 17], and so it is difficult to draw comparisons between our study and theirs. In contrast, Del Rio, in the largest published series of patients undergoing post-thyroidectomy PTH measurement, reported that only 49 of 101 patients developing symptomatic hypocalcaemia were identified by below normal POD1 PTH levels, prompting the authors to abandon postoperative PTH measurement[2]. However, in this study, a lower PTH cut-off was used (12 pg/ml), and POD1 calcium levels were not used in combination.

Limitations of the present study include the inherent subjectivity in the assessment of symptomatic hypocalcaemia. However, we feel that given the prospective data recording as well as the policy to keep all patients in hospital for two postoperative days, we were optimally placed to capture all genuine cases of symptomatic hypocalcaemia. Furthermore, among patients with symptoms, we did not document the severity of symptoms. Thus, it is possible that some patients counted as having symptomatic hypocalcaemia had mild symptoms which might have been self-limiting without treatment. Need for intravenous calcium might be considered a useful surrogate for severe symptomatic hypocalcaemia. However, the decision to administer intravenous calcium can be quite arbitrary, often made at night by junior doctor on call, and there may be a lower threshold to administer calcium to patients who are already in the hospital, when symptoms might have been manageable with oral calcium. Finally, it is uncertain if our results could have been affected by the variability in the timing of POD1 PTH draw, which could range from anything between 12 and 20 h after the surgery. On the other hand, advantages of this study include the large cohort size; the prospective data collection; the fact that all patients were kept in hospital for two nights, which facilitated accurate documentation of symptoms, and the performance of calcium draws on both POD1 and POD2, which facilitated capture of cases of ll as biochemical hypocalcaemia; and the fact that patients were not routinely prescribed oral calcium perioperatively.

## Conclusion

PTH levels performed on POD1 would appear to reliably predict patients at low risk of symptomatic hypocalcaemia. A PTH cut-off of 15 pg/ml, combined with calcium levels ≥ 2.0 mmol/l, can be used for discharge of most patients on POD1 without calcium supplementation, although a higher threshold of 20 pg/ml might be considered for certain patient groups.

## Data Availability

Anonymized data may be provided by the corresponding author on reasonable request.
